# The Anthelmintic—Banksia Abyssinica or Kousso

**Published:** 1851-02

**Authors:** 


*The Anthelmintic—Banksia Abyssinica or Kousso.*—Mr. Vaughn, Port and Civil Surgeon at Aden, Arabia Felix, in a letter to the editor of the London Lancet, on the character of Kousso, says:

Sir—In the Lancet of September, I observe a most interesting report of the cases treated by "Kousso," from a clinical lecture delivered by Dr. Budd, on the 31st of May, in King's College Hospital; and as it appears to be a medicine but recently introduced into Europe, and to be hardly attainable there at times, save at a most exorbitant price, I take the liberty of addressing to you the following brief notice of the plant, a sample of which I hope to be able to forward to Dr. Budd, at King's College Hospital, by the next mail, *viâ* Southampton.

In the report above alluded to, I observe that the "Kousso" is called the "Brayera Anthelmintica," from Dr. Brayer, who first introduced the use of the plant, or rather made its virtue known in Europe. On referring to Bruce's Travels, vol. vii. Appendix, a minute and accurate description of the medicine is found, as also an engraving of the plant in the volume of Plates. That great and much calumniated traveler calls it, in the testimony of esteem for a friend, "Banksia Abyssinica;" and I submit, with all deference, that as James Bruce was the first traveler who brought the plant into notice, the name given by him ought to be retained.

In the appendix to the second volume of the "Highlands of Ethiopia," by the late lamented Sir W. C. Harris, the following mention is made of the "Cossoo," by Dr. Kirk, who was a member of the embassy:—

"'Hagenia Abyssinica' (Cossoo') affords, in a cold infusion of the dried flowers and capsules, the famous drasticum purgans and anthelminticum of the Abyssinians. The tree is one of the most picturesque in appearance."—vol. ii. p. 414.

In Southern Arabia, on the sea cost, and along the shores of the Red Sea, it is well known, and considered a most valuable medicine. In the Northern and Southern Abyssinia it is universally used about once a month, the trees being numerous, and the medicine costing hardly anything. At Hurrur, a province bordering on the kingdom of Shoa, it is well known, and used; though the Somali tribes, residing to the eastward, on the N.E. horn of Africa, are unacquainted with its virtuas. Bruce mentions that no Abyssinian will travel without it; and I am informed by my friend Lieutenant Crutterden, Assistant Political Agent at Aden, that such is actually the case, to his knowledge: the few Abyssinians who visit the port of Aden invariably bring with them a supply. The dose

I understand to be a small handful of the flowers mixed with water, in which sometimes tamarinds are infused, the patient, whilst under its effects, keeping far aloof from every one. Its operation is speedy and most effectual.

In a country like Abyssinia, where almost all, if not the entire population are more or less affected with tapeworm, the "Kousso" is a special blessing of Providence, as a medicine within the reach of the poorest shepherd, and infallible in its effects.

